# Influence of lignin and cellulose from termite-processed biomass on biochar production and evaluation of chromium VI adsorption

**DOI:** 10.1038/s41598-024-65959-5

**Published:** 2024-06-28

**Authors:** Annelise Kopp Alves, Tailane Hauschild, Tania Maria Basegio, Felipe Amorim Berutti

**Affiliations:** Materials Engineering Department, Federal University of Rio Grande do Sul, Av. Osvaldo Aranha, 99/711, Porto Alegre, RS 90035-190 Brazil

**Keywords:** Pyrolysis, Termite, Chromium VI, Adsorption, *Pinuselliottii*, Pollution remediation, Energy

## Abstract

The increasing water contamination by toxic heavy metals, particularly hexavalent chromium, has become a significant environmental concern. This study explores the pyrolysis of termite-processed biomass, specifically *Pinus* *elliottii* particleboard and its termite droppings (TDs), to produce biochar and its application for chromium (VI) adsorption. Termite droppings, rich in lignin, and particleboard, rich in cellulose, were pyrolyzed at various temperatures to assess the effect of biomass composition on biochar properties. The study found that lignin-rich termite droppings produced biochar with higher fixed carbon content and specific surface area than cellulose-rich particleboard biochar. FTIR and Raman spectroscopy revealed significant molecular structure changes during pyrolysis, which influenced the adsorption capabilities of the biochar. Adsorption experiments demonstrated that TD biochar exhibited significantly higher chromium (VI) adsorption capacity, attributed to its distinct chemical composition and enhanced surface properties due to higher lignin content. These findings underscore the crucial role of lignin in producing efficient biochar for heavy metal adsorption, highlighting the practical applicability of termite-processed biomass in water purification technologies.

## Introduction

The most abundant polysaccharide in nature is cellulose, a linear polymer of d-glucosyl units surrounded by hemicellulose and lignin, connected by intra/intermolecular hydrogen bonds and by β-1,4-glycoside^1^. Due to its relatively simple structure, cellulose has been widely applied in pyrolysis. Comparatively, hemicellulose has a more complex structure with many branched chains and substituents. It is primarily composed of xylan and glucomannans, which possess the pyrolytic properties of anhydrosugars, resulting in pyrolytic characteristics similar to cellulose^2^. The pyrolysis process of xylan can be used to study the pyrolytic mechanism of hemicellulose, including initial depolymerization, the generation of furan and pyran derivatives via dehydration, and the breakage of furanose and pyranose-derived rings to form light oxygenates^2^.

Lignin is a complex biopolymer with an aromatic structure, mainly associated with cellulose and hemicellulose, to form plant skeletons. Higher temperatures are necessary for chemical bond cleavage during lignin pyrolysis, producing large amounts of phenolic compounds, along with acids, alcohols, and light aromatic hydrocarbons^3^.

In nature, cellulose and hemicellulose are encrusted with lignin, which protects against enzymatic attack in lignocellulosic materials. However, wood-feeding termites (WFTs) can efficiently digest lignocellulosic substrates^4^. Wood-cellulose digestion by WFTs is likely facilitated by cellulases and pretreatment factors that modify lignin and enhance cellulose accessibility. Structural modification of lignin appears crucial for deconstructing the plant cell wall and utilizing cellulose. However, the precise mechanism by which WFTs overcome the lignin barrier and enhance cellulose accessibility remains unclear^4,5^. Termites consume an estimated 3 to 7 billion tons of lignocellulosic materials each year, making them among the most prolific and efficient decomposers of lignocellulose worldwide^6^. Termite droppings contain more than 50% lignin and can be considered an abundant source of biomass for use as a low-cost adsorbent^7^.

Biochar is a carbon-rich product obtained from the thermal decomposition of organic material in the presence of little or no oxygen (pyrolysis)^8^. It is not a single substance but a group of substances with a broad range of properties^9^. Consequently, biochar can be used for various applications, such as an adsorbent, soil improver, additive in animal feed, activated carbon, construction material additive, and catalyst or catalyst support^10,11^.

Pyrolysis has emerged as a suitable method for converting various feedstocks, including biomass and solid wastes, into energy vectors (e.g., charcoal, biofuels) or organic products (biochar, bio-oils)^12^. However, the properties of biochar depend on its feedstock and production process^9^. The pyrolysis type, temperature, heating rate, and duration can also drastically affect the quality of the resulting biochar^13^. The organic composition, particularly the amounts of lignin and cellulose, affects the quantity and quality of the pyrolytic products^14^.

In this context, this work aims to (i) evaluate the interplay of lignin and cellulose in the pyrolysis process of *Pinus* *elliottii* particleboards and its termite droppings; (ii) examine the effect of the lignin/cellulose ratio on the type of pyrolytic product obtained (biochar/gas/oil); and (iii) evaluate the application of the different biochars obtained in the absorption of hexavalent chromium in water.

## Materials and methods

Particleboards of *P.* *elliottii* (500 × 900 × 20 mm) were fed to dry-wood termites (*Cryptotermes* brevis). An original particleboard sample was collected for comparison purposes and prepared using a knife mill (Solab, SL32), with the particles passing through a sieve with an opening of 0.177 mm (Mesh 80). The termite droppings (TDs), the residue of digested particleboards, were collected after a year of termite attacks on the wood board.

### Hemicellulose, cellulose, and lignin content

The hemicellulose, cellulose, and lignin content of the particleboard and the termite-dropping samples were determined using the Van Soest Method^15^. This method is based on the analysis of ADF (Acid Detergent Fiber, which includes cellulose and lignin), NDF (Neutral Detergent Fiber, which includes hemicellulose, cellulose, and lignin), and ADL (Acid Detergent Lignin, which measures lignin content).

### Pyrolysis

Biomass pyrolysis in an inert (nitrogen) atmosphere was used to produce biochar from the milled original particleboard and termite droppings. The biochar was obtained using 20 g of the biomass in a horizontal tubular quartz furnace at temperatures of 450, 550, and 650 °C, with a heating rate of 10 °C min^−1^, under nitrogen flow (250 cm^3^_NPT_ min^−1^) to prevent the entry of O_2_ and to remove the pyrolysis gases released during the process. The soak time for all experiments was 30 min. After pyrolysis, the biochar was collected from the reactor and saved for subsequent characterizations.

### Proximate analysis

The proximate analysis, widely used as the basis for coal characterization, is presented as a group of test methods that separates the products into four groups: (1) moisture (SIS-CEN/TS 14774-1:2004); (2) volatile matter (SIS-CEN/TS 15148:2006), consisting of gases and vapors released during pyrolysis; (3) fixed carbon (SIS-CEN/TS 14588:2003), the nonvolatile fraction of coal; and (4) ash content (SIS-CEN/TS 14775:2004), the inorganic residue remaining after combustion.

### Thermal behavior

Thermogravimetric analysis (TGA) was used to determine termite-induced changes in the general characteristics of lignocellulose decomposition under pyrolytic conditions. TGA was conducted using Shimadzu TG-50 equipment. Approximately 5 mg sample was loaded into a platinum pan and heated from 25 to 700 °C at a heating rate of 10 °C min^−1^ under a nitrogen atmosphere with a flow rate of 50 mL min^−1^.

### Functional groups, composition, and morphology

The Fourier Infrared (FTIR) analysis (IRAffinity-1, Shimadzu) using the ATR method, with samples diluted in KBr, was used to evaluate the functional groups and their changes during the pyrolysis process. Raman spectroscopy (InVia, Renishaw) was employed to assess the carbonaceous characteristics of the samples. Scanning electron microscopy (SEM, EVOMA10, Carl Zeiss) was used to examine the morphology of the particleboard and termite droppings before and after the pyrolysis at different temperatures. N_2_ adsorption/desorption isotherm analysis (Nova 1000e, Quantachrome) was performed to evaluate the porosity (by DFT method) and specific surface area (SSA, using the BET method). For this analysis, 0.1 g of each sample was dried at 100 °C for 24 h in an oven and 2 h at 300 °C in vacuum.

### Determination of pH and pH_pzc_ in biochar

The pH of biochar was determined using a method for testing the pH of waste materials (EN 12457-2:2002). The point of zero charge (pH_pzc_) was used to characterize the surface acidity and alkalinity of the activated carbon^16^.

### Adsorption isotherm

Adsorption isotherm models were employed to examine the relationship between the mass of the biochar used and the amount of chromium (VI) adsorbed at equilibrium, with chromium concentrations ranging from 40 to 100 mg L^−1^. These models were also utilized to elucidate the interaction between chromium adsorbent molecules and surface adsorption sites. The optimal conditions maintained were an adsorbent dose of 0.5 g/100 mL, a solution pH of 5.5, and a contact time of 10 h under shaking. After that time, the biochar system reached equilibrium concentration. Then, the solutions were centrifuged, and the chromium (VI) concentrations were determined using a UV–vis spectrophotometer (Cary 7000, Agilent) at a wavelength of 540 nm, employing 1,5-diphenylcarbazide as an indicator.

The Langmuir and Freundlich isotherm models were applied to evaluate the fitness of the data and to determine whether the adsorbent surfaces were homogeneous or heterogeneous^16^. The Langmuir isotherm model assumes that adsorbates form a monolayer on binding sites. The Freundlich isotherm model assumes that the entire biochar surface is multilayered during adsorption. The equations used are presented in Table 1.

**Table 1:** Langmuir and Freundlich models of equilibrium adsorption and sorption capacity.

Model	Equation	Symbol explanation
Langmuir	$\frac{{C}_{eq}}{{q}_{eq}}=\frac{{C}_{eq}}{{q}_{max}}+\frac{1}{{K}_{L}*{q}_{max}}$	*q*_*eq*_ is the amount adsorbed by chromium (VI) at equilibrium, mg g^−1^ *C*_*eq*_ is the the equilibrium concentration in solution, mg L^−1^ *q*_*max*_ is the monolayer capacity of the adsorbent, mg g^−1^ *K*_*L*_ is the Langmuir adsorption constant, L mg^−1^
*R*_*L*_	${R}_{L}=\frac{1}{1+{C}_{0}*{K}_{L}}$	*R*_*L*_ is the dimensionless *C*_*0*_ is the initial concentration in solution, mg L^−1^ *K*_*L*_ is the Langmuir adsorption constant, L mg^−1^
Freundlich	$\text{ln}\left({q}_{eq}\right)=\text{ln}\left({K}_{F}\right)+\frac{1}{n}\text{ln}\left({C}_{eq}\right)$	*q*_*eq*_ is the amount adsorbed by biochar at equilibrium, mg g^−1^ *C*_*eq*_ is the equilibrium concentration in solution, mg L^−1^ *K*_*F*_ is the Freundlich constant, mg^1−1/n^ L^1/n^ g^−1^ *1/n* is the heterogeneity factor, –

### Adsorption kinetics

For kinetic experiments, the biochar obtained from particleboard and TD pyrolyzed at different temperatures (50 mg) were mixed with 50 mL aqueous K_2_CrO_4_ solution [200 mg L^−1^, chromium (VI)] at an initial pH of 5.5. The mixtures were shaken for 0.5, 1, 2, 3, 5, 12, 18, 24, 30, 36, 42, and 48 h. All experiments were conducted at 25 °C.

The adsorption capacities of the biochar were determined using Eq. (1):

1 $${q}_{e}=\frac{\left({C}_{0}-{C}_{e}\right)V}{M}$$

where q_e_ (mg g^−1^) is the adsorption capacities of the biochar; C_0_ (mg L^−1^) and C_e_ (mg L^−1^) are the chromium (VI) concentrations before and after adsorption, respectively; V (L) is the volume of the adsorbate solution; M (g) is the amount of biochar.

The adsorption kinetics was described by the pseudo-first-order (Eq. 2) and pseudo-second-order (Eq. 3) models^17^:

2 $$\text{ln }\left({q}_{e}-{q}_{t}\right)= {ln q}_{e}-{k}_{1}t$$

3 $$\frac{1}{{q}_{t}}=\frac{1}{{k}_{2}{q}_{e}^{2}}+\frac{1}{{q}_{e}}t$$

where q_e_ (mg g^−1^) is the equilibrium capacity, q_t_ (mg g^−1^) is the instant adsorption capacity, and k_1_ (h^−1^) and k_2_ (g·mg^−1^·h^−1^) are the corresponding adsorption rate constants.

## Results and discussion

### Biomass characterization and pyrolysis

Table 2 indicates the lignocellulosic composition of *P.* *elliottii* particleboard and its termite droppings.

**Table 2:** Hemicellulose, cellulose, and lignin content of undigested particleboard and in the termite droppings.

	Particleboard	Termite droppings
Hemicellulose (wt.%)	13.2	12.6
Cellulose (wt.%)	47.6	11.8
Lignin (wt.%)	25.0	52.6

The cellulose content of the biomass was reduced by 75% due to termite activity. These changes resulted in the TDs being richer in lignin, comprising 52.6% of its mass, doubling the relative amount of this compound compared to the original particleboard, which contained 25% of its mass. Ke et al.^18^ also determined that the lignin content in termite droppings was concentrated compared with the undigested wood. Using chromatographic techniques, they revealed that the relative number of lignin-derived components increased in the termite droppings. These findings support the idea that during the cell-wall degradation process and hydrolysis of cellulose by termites, the native lignin macromolecular assembly undergoes structural modification while conserving the interunit lignin linkage and retaining the original aromatic characteristics.

Interestingly, there is a slight decrease in the hemicellulose content (approximately 4.5%). Hemicellulose has a more complex structure than cellulose, with abundant branched chains and substituents, which can be difficult for termite enzymes to digest.

**Figure 1:**
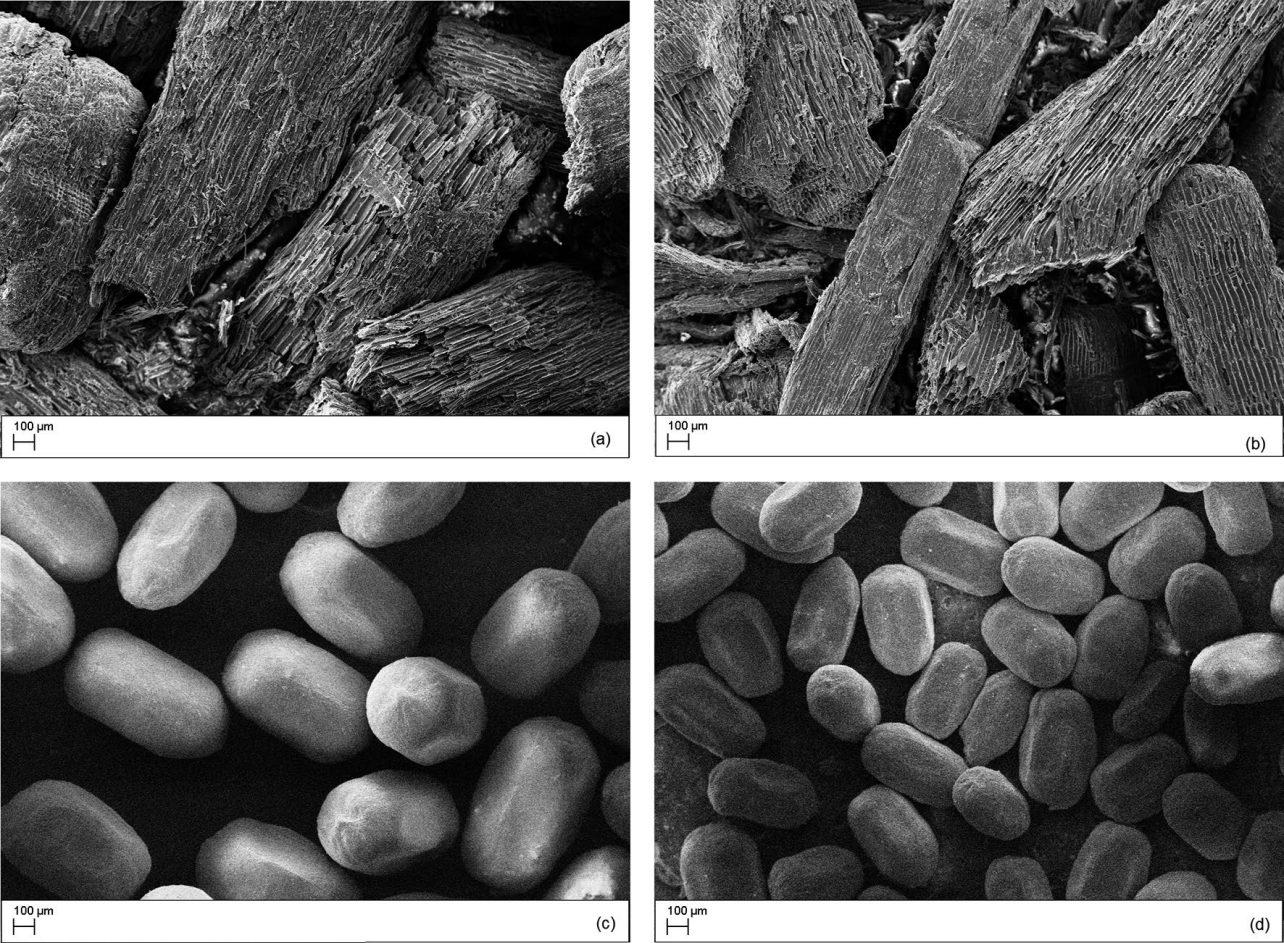
SEM images of particleboard chips and termite droppings (TDs) before (**a,c**) and after pyrolysis at 650 °C (**b,d**), respectively.

Figure 1a, b present the morphology of the *P.* *elliottii* particleboard chips before and after the pyrolytic process at 650 °C, respectively. Figure 1c, d show the morphology of TDs in the same condition.

The particleboard chips appear elongated with varying lengths and diameters, exhibiting a plicated surface and irregular small pieces typical of knife-milled wood chips. The wood channel and wall structure were maintained after pyrolysis at 650 °C and are more evident after this treatment, likely due to the partial decomposition of the organic fraction.

The termite droppings also maintain their shape after pyrolysis. They are oval-shaped with six concave sides, exhibiting a smooth and homogeneous surface without evidence of large pores. Before the pyrolytic treatment, the average length of the TDs was 708.59 μm, and their width was 416.3 μm. The pyrolysis at 650 °C reduced the length and diameter by approximately 28%.

**Figure 2:**
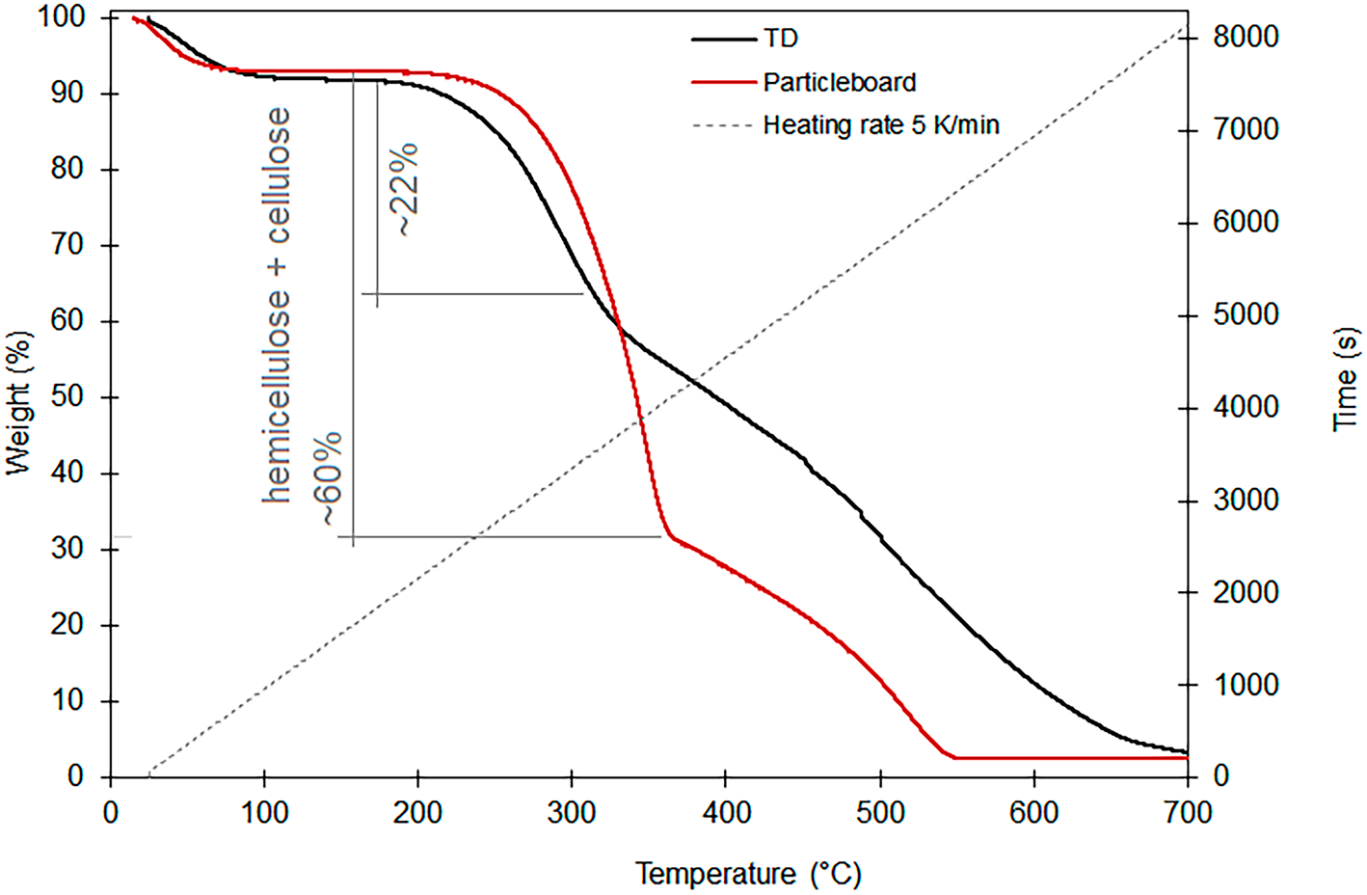
Thermogravimetric analysis results (nitrogen atmosphere) of particleboard and termite-dropping (TD) samples.

The TGA curves under the nitrogen atmosphere for both the particleboard and TDs are in Fig. 2. Considering that the TDs are rich in lignin, the comparative results indicate that the overall reaction rate decreases with the increase of the lignin content.

While comparing the thermal decomposition behavior of the TD to the particleboard, the TGA curve displayed fluctuations during the biomass decomposition. TD decomposition began earlier, and the maximum rate shifted to the left compared to the particleboard sample, indicating that a lower temperature can convert the TD sample to volatiles. The lowering of the initiation temperature corresponds to a decrease in the minimum energy required to start the active gasification reaction in the lignin-rich TD. Meanwhile, the maximum rate of thermal decomposition of the TDs decreased, indicating a lower amount of cellulose and/or hemicellulose^19^.

Initially, both materials exhibited mass loss of up to about 100 °C due to the loss of around 9% adsorbed water. The proximate analysis (Table 3) indicates that both samples have similar moisture content: 9.12% for the particleboard and 9.70% for the TDs.

**Table 3:** Specific surface area (SSA), total pore volume, average pore diameter, moisture, volatile matter, ash, fixed carbon content, and biochar yield of *Pinus* *elliottii* particleboard and termite-dropping samples.

	SSA (m^2^ g^−1^)	Total pore volume (cm^3^ g^-1^)	Average pore diameter (Ǻ)	Moisture (% wt.)	Volatile matter (% wt.)	Ash (% wt.)	Fixed carbon (% wt.)	Biochar yield (% wt.)
Particleboard (Pb)	2.9	0.03	2.0	9.12 ± 0.13	81.93 ± 0.12	0.87 ± 0.08	17.2 ± 0.16	–
Pb-450	3.0	0.03	2.1	7.58 ± 0.37	26.88 ± 0.44	1.03 ± 0.08	72.1 ± 0.50	28.48 ± 0.01
Pb-550	14.9	0.09	15.4	7.33 ± 0.19	23.71 ± 0.77	3.66 ± 0.38	72.6 ± 0.8	25.75 ± 0.02
Pb-650	78.5	0.17	30.1	7.45 ± 0.17	21.11 ± 1.38	5.80 ± 0.20	73.1 ± 0.8	25.16 ± 0.05
Termite droppings (TDs)	3.8	0.03	2.1	9.70 ± 0.10	68.61 ± 0.57	2.77 ± 0.01	28.62 ± 0.56	–
TD-450	6.0	0.03	3.0	9.63 ± 0.27	31.47 ± 0.16	7.19 ± 0.25	61.3 ± 0.1	40.77 ± 0.03
TD-550	22.8	0.15	21.4	8.58 ± 0.11	29.95 ± 0.59	8.35 ± 0.94	61.7 ± 0.7	36.50 ± 0.04
TD-650	100.8	0.20	50.5	8.02 ± 0.12	22.23 ± 0.32	9.36 ± 0.25	68.4 ± 0.5	35.44 ± 0.01

Hemicellulose starts its decomposition easily, and the weight loss mainly occurs at 203–386 °C. Meanwhile, cellulose pyrolysis happens at a higher temperature range (286–426 °C), with the maximum weight loss at around 330 °C. Usually, cellulose has a small solid residue (below 4%) because most is volatilized at around 700 °C^20^. Cellulose is depolymerized at around 350 °C, followed by further conversion via bond cracking and dehydration into levoglucosan (LGA), levoglucone (LGO), and other monosaccharides. The primary pyrolytic products from cellulose are anhydrosugars, which can be further converted at higher temperatures into light oxygenates (e.g., furans, aldehydes, ketones, acids, etc.)^21,22^.

The TGA data for the amount of hemicellulose and cellulose perfectly agree with the results from the Van Soest method (Table 2). In both analyses, the sum of these compounds in the particleboard was around 60%, while for the TDs, it was around 20%.

Among the three components, lignin is the most difficult to decompose compared to cellulose and hemicellulose, which exhibit higher weight loss. The behavior of the TD sample is typical of lignin-rich biomass in that the principal decomposition of lignin happened in a wide temperature range from around 300 to 550 °C, where it lost about 60% of its relative weight.

The TGA curves showed a larger residual amount of biomass in the TD sample at temperatures between 400 and 600 °C compared to the particleboard sample. A possible explanation is that once lignin is modified by chewing, part of its intermediate fragments will be rearranged through condensation and re-polymerization, leading to new structures with more stability.

The proximate analysis (Table 3) indicates that the ash content is 21% higher for TDs than the particleboard. This is because termites, through their feeding habits, often incorporate inorganic particles (dust, soil, and other minerals) into their droppings, resulting in a composite material that contains both organic and inorganic components. Additionally, with the consumption of cellulose and hemicellulose, the inorganics increase as they are not digested or adsorbed. The higher ash concentration could promote biochar yields, as inorganic elements in the ash are known to catalyze the formation of solid products during pyrolysis^23^.

The TGA curve for the TDs, which is rich in lignin, evidences that the thermal mass loss is less pronounced than the particleboard curve. Lignin, being a highly cross-linked and three-dimensional polymer, can influence the pyrolysis process by increasing the heat resistance and decelerating the degradation rate of the biomass. Its intricate and heterogeneous chemical structure results in biochar with higher fixed carbon content and greater surface area than feedstocks with lower lignin content. The TDs, rich in lignin, exhibit a fixed carbon content 60% higher than the undigested particleboard and a higher surface area for the studied pyrolysis temperatures (Table 3).

Hemicellulose and cellulose, on the other hand, are simpler polymers that readily degrade during the pyrolysis process and can serve as a source of volatile organic compounds, presenting lower fixed carbon content. The *P.* *elliottii* particleboard, with 75% more cellulose than the TDs rich in lignin, had approximately 13% more volatile compounds than the TDs (Table 3).

Moreover, based on a comparison of volatile matter and fixed carbon, the yields of pyrolytic products can be predicted to be significantly different for the particleboard and TD biomasses. Considering the weights of the initial biomass and solid content after pyrolysis, the biochar yield indicates higher values, around 41%, for the lignin-rich TDs for each treatment temperature (Table 3). The particleboard, richer in hemicellulose and cellulose, contained the highest volatile matter (81.9%) and could be expected to yield the highest amounts of volatile products. After pyrolysis, the biochar obtained from particleboard (cellulose-rich) has around 24% of volatile matter remaining in the structure, indicating a yield of about 58 wt. % in volatiles. The lignin-rich TDs have a higher fixed carbon content (around 63% after pyrolysis) and can lead to higher biochar yields, as lignin has a complex structure and poses more resistance to thermal degradation than holocellulose (cellulose with hemicellulose), also due to its high level of aromaticity, size, and structural arrangement, which affects the proportion of the solid product generated^24^. Moreover, the decrease in biochar yield is due to increased pyrolysis temperature, probably from the thermal cracking of volatile components into lower molecular weight liquids and gases rather than biochar^25^.

**Figure 3:**
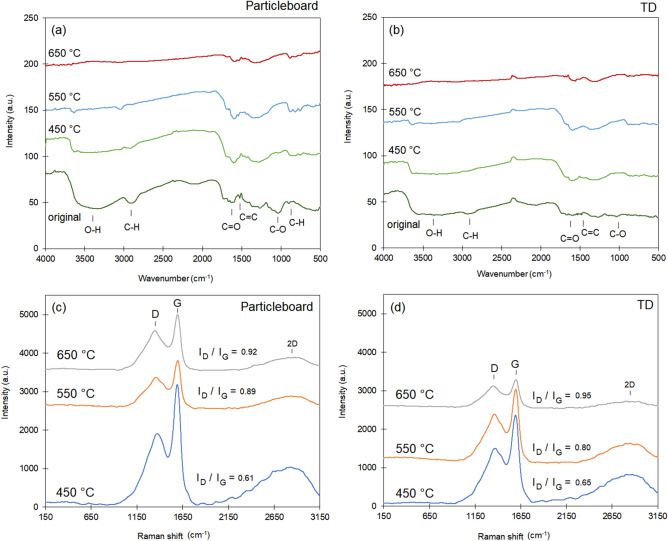
Results of the FTIR and Raman analysis of the (**a,c**) particleboard chips and (**b,d**) termite droppings (TDs) after the pyrolysis at different temperatures.

Results obtained from FTIR spectra for the particleboard biochar demonstrated an increase in aromatic groups and a decline in acidic groups with increasing pyrolysis temperature (Fig. 3a). Biochar from particleboard pyrolyzed from 450 to 650 °C exhibited a decrease in relative intensity of the following bands as the temperature increased: 3500 cm^−1^ (O–H stretching of hydroxyl groups), 2900 cm^−1^ (C–H asymmetric and symmetric stretching of aliphatic groups), 1620 cm^−1^ (C=O stretching of carboxyl mode), and 1026 cm^−1^ (C–O symmetric stretching associated with cellulose, hemicellulose, and lignin). Other bands demonstrated an increase in relative intensity of around 900–800 cm^−1^ (C–H aromatic deformation modes), 1600 cm^−1^ (C=C aromatic stretching and C=O stretching of conjugated ketones and quinones), and 1400 cm^−1^ (C–C stretching of aromatic rings)^26^.

The termite-dropping FTIR spectra bands (Fig. 3b) were much more subtle than the particleboard ones. Up to 550 °C, few little alterations occurred in the intensity of functional groups. However, a more significant increase in the intensity was observed around 1600 cm^−1^ (C=C aromatic stretching and C=O stretching of conjugated ketones and quinones) with increasing pyrolysis temperatures. Since TDs were richer in lignin, a more complex biomass, the pyrolysis reaction was slower and occurred at a higher temperature. Consequently, the formation of byproducts took longer and required more energy. This slower reaction rate was likely the cause of the subtle changes observed in the FTIR spectra of this material.

The Raman spectra of the biochar obtained from particleboard chips (Fig. 3c) and TDs (Fig. 3d) at different pyrolysis temperatures helped elucidate the formation of carbon structures. For both types of biochar, the position of the D band (around 1400 cm^−1^) shifted toward a lower wavenumber for higher temperatures, while the position of the G band (around 1600 cm^−1^) remained constant, indicating the formation of larger aromatic ring clusters^27^. The I_D_/I_G_ ratios increased, and the full width at half maximum (FWHMD) decreased with higher temperatures. This indicated greater structural order and the formation of larger aromatic ring clusters^19^.

In addition to the chemical composition, the porosity, including specific surface area (SSA) and micro-mesopore structures, are fundamental characteristics that significantly influence the efficacy of sorbents in the adsorption process. Both particleboard and TDs displayed greater SSA following the temperature increase (Table 3), attributed to the significant degree of organic matter decomposition associated with volatile release and the subsequent formation of a porous structure. Lignin is known to be more thermally stable than cellulose. Consequently, during pyrolysis, lignin tends to undergo decomposition over a broader range and at higher temperatures than cellulose (Fig. 2). As a result, the pyrolysis process for lignin-rich TD may involve more prolonged heating or higher temperatures to achieve decomposition, forming a more complex carbonaceous structure with a higher surface area and mesoporosity. Table 3 indicates that with the increase in SSA, the total pore diameter and the average pore diameter increased for both particleboards and TD samples.

These results have demonstrated that changes in lignin content have altered the surface physical properties of the biochar, which can positively influence its adsorption properties.

### Chromium (VI) adsorption

#### Adsorption isotherm

The parameters of the Langmuir and Freundlich isotherm models are listed in Table 4.

**Table 4:** Parameters of the Langmuir and Freundlich isotherm models.

Adsorbent	Langmuir			Freundlich		
	q_max_ (mg g^−1^)	b (L mg^−1^)	R^2^	k_f_ (mg g^−1^)	1/n	R^2^
PB 450	28.4	0.45	0.99	6.6	0.12	0.82
PB 550	43.6	0.03	0.97	7.8	0.32	0.97
PB 650	51.0	0.12	0.97	10.2	0.16	0.97
TD 450	60.2	0.03	0.93	6.1	0.41	0.97
TD 550	62.4	0.09	0.97	10.6	0.35	0.90
TD 650	71.0	0.08	0.94	12.4	0.35	0.90

The data indicate that chromium (VI) adsorption onto particleboard and TD biochars fits both the Langmuir (R^2^ values of 0.93–0.99) and Freundlich models (R^2^ values of 0.82–0.97), suggesting the involvement of both chemical and physical adsorption mechanisms. Biochars pyrolyzed at 650 °C exhibited the highest adsorption capacities for chromium (VI), with maximum adsorption capacities calculated by the Langmuir isotherm being 71.0 mg g^−1^ and 51.0 mg g^−1^ for TD and particleboard biochars, respectively.

According to the Langmuir model, the dimensionless constant separation factor (R_L_) can predict whether an adsorption system is favorable or unfavorable. The R_L_ value indicates the type of isotherm: unfavorable (R_L_ > 1), linear (R_L_ = 1), favorable (0 < R_L_ < 1), or irreversible (R_L_ = 0). The R_L_ values ranged from 0 to 1, indicating that adsorption of chromium (VI) on these biochars was favorable under the experimental conditions, demonstrating a high affinity for chromium (VI).

#### Adsorption kinetics

**Figure 4:**
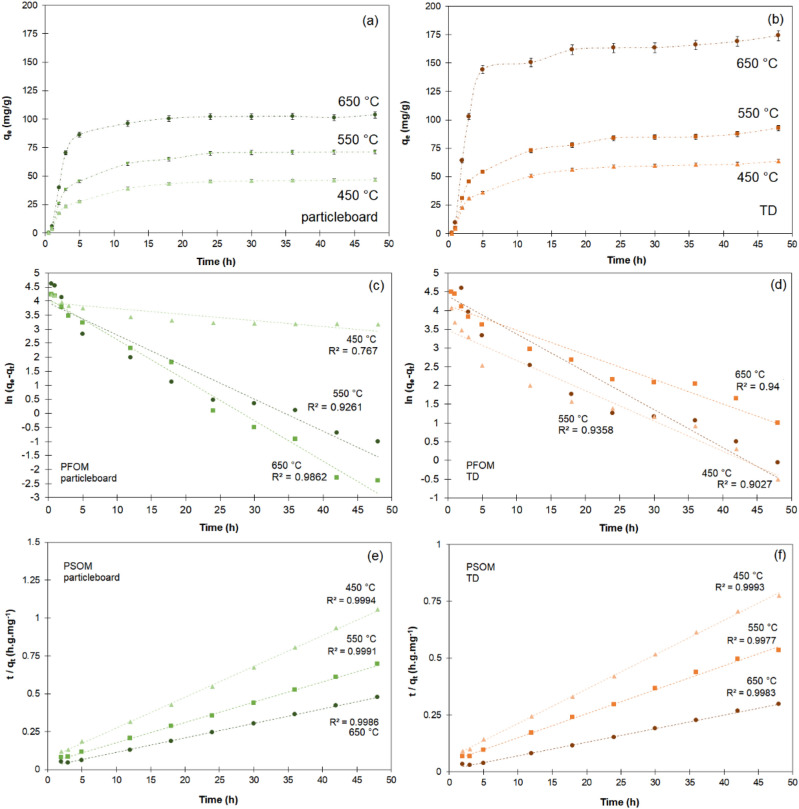
Adsorption kinetics of biochar (**a**) particleboard, (**b**) termite droppings (TDs) for different pyrolysis temperatures. Pseudo-first-order model (PFOM) and pseudo-second-order model (PSOM) for the adsorption kinetics of Cr (VI) over biochar obtained from (**c,e**) particleboard, (**d,f**) termite droppings for different pyrolysis temperatures.

For the chromium (VI) adsorption capability of the biochar, the adsorption increased with the higher pyrolysis temperature for both types of biochar (Fig. 4). Initially, the absorption capability rose, but after approximately 24 h, a plateau was reached. The smooth and continuous nature of the curves indicates monolayer coverage of chromium (VI) on the surface of the biochar^28^.

The best adsorption capacity for the particleboard chips was 100.66 mg g^−1^; for the TDs, it was 169.1 mg g^−1^, both for samples heat-treated at 650 °C. These results can be directly associated with the SSA of the particles at different pyrolysis temperatures. For both particleboards and TDs, the SSA increased with the increase of the pyrolysis temperature (Table 3). This is likely due to the formation of more mesopores caused by the escape of volatile substances as the temperature increased^29,30^. According to Chen et al. (2012)^31^, a higher surface area and porosity of biochar are mainly associated with the decomposition of lignin, the rapid release of H_2_ and CH_4_, and the reaction of aromatic condensation as the temperature rises.

The surface characteristics of biochar are crucial when considering its application as an adsorption agent in water systems. At lower pyrolysis temperatures, up to 650 °C, lignin is not entirely converted into hydrophobic polycyclic aromatic hydrocarbon (PAHs), making biochar more hydrophilic. At temperatures higher than 650 °C, biochar becomes thermally stable and more hydrophobic^32^.

Analysis of the FTIR spectra of both types of biochar (Fig. 3a, b) indicates that the aromatic groups are more pronounced in the particleboard samples. In contrast, the TDs do not show significant stretching bands for these compounds, possibly due to their lower content. This suggests that, in general, the TD samples can be considered more hydrophilic. Coupled with a higher surface area, this hydrophilicity resulted in a higher adsorption rate.

The main functional groups found in biochar that may contribute to the adsorption process include (i) aromatic rings; (ii) O–H groups, possibly from alcohols or phenols; (iii) C=O groups, possibly from carboxylic acids and esters; and (iv) C–H groups from aromatic and aliphatic compounds. These functional groups enhance the adsorption properties of biochar, making it effective in removing contaminants from water.

Independent of the temperature treatment, the pH of the biochar obtained from the particleboards was 6.0, and the pH of the TD biochar was 8.0. Pyrolysis studies indicate that higher temperatures lead to a progressive concentration of the inorganic constituents that comprise ash^33^, thus increasing the pH of the obtained biochar. The TD biochar exhibited a higher ash content at all pyrolysis temperatures. At 650 °C, the ash content of the TD biochar is 1.6 times that of particleboard biochar (Table 3). The pH_pzc_ was utilized to assess the surface charge of the produced biochar. The TD biochar had a pH_pzc_ of 7.2, and the particleboard biochar had a pH_pzc_ of 6.0. Determining the point of zero charge in biochar is crucial for understanding its surface charge properties. At pHpzc, the net surface charge of the biochar is neutral, indicating an equal concentration of positive and negative charges on its surface. Above pH_pzc_, the biochar surface becomes negatively charged, attracting positively charged ions.

Conversely, below pH_pzc_, the surface carries a positive charge, thereby attracting negatively charged ions. For chromium (VI) adsorption, a solution of K_2_CrO_4_ was employed at pH 5.5. Under these conditions, the potential adsorption ions are Cr_2_O_7_^2−^ and, eventually, CrO_4_^2−^, which can consequently be attracted to the biochar surface.

The results of pseudo-first-order and pseudo-second-order rate equations for the particleboard chips and TDs are presented in Fig. 4c, d and e, f, respectively, and Table 5.

**Table 5:** Kinetic parameters of chromium (VI) adsorption on particleboard (PB) and the termite droppings (TDs) after pyrolysis at different temperatures.

Kinetic model	Parameters	PB 450	PB 550	PB 650	TD 450	TD 550	TD 650
PFOM	*q*_*e*_ (mg g^−1^)	69.1	69.1	100.7	62.0	162.0	90.2
	*k*_1_(h^−1^)	0.0210	0.1144	0.1144	0.1009	0.0809	0.0657
	*R*^2^	0.7670	0.9261	0.9862	0.9027	0.9358	0.9027
PSOM	*q*_*e*_ (mg g^−1^)	49.3	74.6	105.3	66.2	94.3	166.7
	*k*_2_ (g mg^−1^ h^−1^)	0.006	0.004	0.005	0.004	0.003	0.003
	*R*^2^	0.9994	0.9991	0.9986	0.9993	0.9977	0.9983

The kinetic parameters at the equilibrium state q_e_ and adsorption constant K_1_ calculated from the pseudo-first-order model (PFOM) are indicated in Table 5. PFOM is based on the premise that the rate of solute uptake change over time is directly proportional to the difference between the saturation concentration and the amount of solute uptake by the solid over time. Adsorption kinetics frequently adhere to the Lagergren pseudo-first-order rate equation when adsorption occurs via diffusion through the interface. However, the outcomes obtained in this study are inconsistent with the experimental results and have low correlation coefficients (R^2^), indicating that this model is unsuitable for chromium adsorption (VI).

The pseudo-second-order model (PSOM) assumes that chemisorption is the rate-determining factor for adsorption, estimating the behavior across the entire adsorption spectrum. Under these circumstances, the adsorption rate depends on the adsorption capacity rather than the adsorbate concentration. In this work, the correlation coefficients (R^2^) of PSOM were higher (over 0.99) than those of PFOM, thus supporting that the adsorption of chromium (VI) by the obtained biochar was a chemisorption process. As described by the pseudo-second-order model, the adsorption kinetics seems reasonable since it takes longer to reach equilibrium, with the limiting step being chemisorption. This indicates that the rate is determined by the interaction with surface sites, which aligns well with the observation that the adsorption rate was higher for the higher SSA lignin-rich TD samples.

#### Hexavalent chromium adsorption mechanisms

Based on the results obtained, four adsorption mechanisms for chromium (VI) using biochar derived from *P.* *elliottii* and its termite droppings can be proposed:

(i) Surface adsorption: the adsorption of hexavalent chromium from potassium chromate (K_2_CrO_4_) solutions onto biochar primarily involves surface interactions between the chromium VI ions and the biochar. The biochar produced from termite-processed biomass and *P.* *elliottii* particleboard exhibits various functional groups (hydroxyl, carboxyl, and phenolic groups) crucial for adsorption^34^. Additionally, the large surface area of the biochar facilitates physical adsorption, enhancing the overall adsorption capacity.
(ii) Electrostatic interaction: chromium VI exists as chromate ions (CrO_4_^2−^) in aqueous solutions. These negatively charged ions can be attracted to the positively charged sites on the biochar surface, facilitating their adsorption^35^.
(iii) Ion exchange: the functional groups on the biochar surface can exchange ions with the chromate ions^34^.
(iv) Complexation: complexation is a process in which electrons interact with donors and acceptors to form various complexes. Functional groups on the biochar surface can form complexes with chromium (VI) ions, further contributing to the adsorption process^36^.

These interactions highlight the role of biochar surface chemistry in chromium (VI) adsorption. The distinct chemical compositions and surface properties of biochar derived from termite droppings and particleboard at different pyrolysis temperatures resulted in different adsorption capacities, with termite-dropping biochar typically showing higher efficiency due to its higher fixed carbon content and surface area.

## Conclusions

This study investigates the pyrolysis of *P.* *elliottii* particleboard chips and termite-digested droppings, focusing on cellulose and lignin content. Termite activity reduces cellulose by 75%. Pyrolysis at different temperatures produced higher biochar yields at lower temperatures and higher lignin content, reaching 40.8% at 450 °C for TD samples. Lignin-rich TD decomposed earlier, influencing biochar properties, resulting in a biochar with 60% fixed carbon and a specific surface area of 100.8 m^2^ g^−1^. Biochar derived from lignin-rich TDs also exhibited superior chromium (VI) adsorption (70% more than cellulose-rich particleboards) due to higher surface area and pH, making it a more effective adsorbent.

## Data Availability

The data that support the outcomes of this study are available from the corresponding author upon reasonable request.
